# High adherence to oral daily PrEP in a real-world implementation project in Kenya: a brief communication

**DOI:** 10.1186/s12981-026-00875-4

**Published:** 2026-03-24

**Authors:** Ivan Segawa, Linxuan Wu, Josephine Odoyo, Margaret Mwangi, Jennifer F. Morton, Elizabeth Irungu, Kenneth Ngure, Nelly R. Mugo, Elizabeth Bukusi, Kenneth K. Mugwanya

**Affiliations:** 1 Clinical Epidemiology Unit, Makerere University, Kampala, Uganda; 2 Department of Epidemiology, University of Washington, Seattle, USA; 3 Department of Global Health, University of Washington, Seattle, USA; 4 Centre for Microbiology Research, Kenya Medical Research Institute, Nairobi, Kenya; 5 Centre for Clinical Research, Kenya Medical Research Institute, Nairobi, Kenya; 6 Jhpiego, Nairobi, Kenya; 7 School of Public Health, Jomo Kenyatta University of Agriculture and Technology, Nairobi, Kenya; 8 Department of Obstetrics and Gynecology, University of Washington, Seattle, USA

**Keywords:** PrEP, Dried Blood Spots, HIV, Kenya, Adherence

## Abstract

Objective biomarkers of oral pre-exposure prophylaxis (PrEP), such as tenofovir diphosphate (TFV-DP) in dried blood spots (DBS), remain critical for interpreting PrEP adherence. We tested 195 DBS randomly collected from 168 of 4,955 PrEP clients returning for refills at 25 public HIV clinics in Kenya. DBS were extracted using the 50:50 methanol: water method, and drug concentrations quantified using liquid chromatography-tandem mass spectrometry. Optimal adherence was defined as ≥ 900 fmol/punch, corresponding to ≥ 4 doses/week. TFV-DP was detectable in 96% (188/195) of samples; 71% (138/195) had optimal adherence, higher among older clients and men. Optimal adherence is achievable in real-world PrEP programs.

**Supplementary Information:**

The online version contains supplementary material available at 10.1186/s12981-026-00875-4.

## Introduction

Oral pre-exposure prophylaxis (PrEP) containing tenofovir disoproxil fumarate and emtricitabine (TDF/FTC) reduces the risk of HIV acquisition by 90% among individuals with detectable drug concentrations [1]. Although adherence assessments in African PrEP programs have relied on self-report, pill counts, or pharmacy refill data, these measures are limited by recall and social desirability biases that often overestimate adherence [2, 3]. Objective pharmacologic adherence metrics, such as tenofovir diphosphate (TFV-DP) and emtricitabine triphosphate (FTC-TP) concentrations measured in dried blood spots (DBS), overcome these biases by directly reflecting cumulative and recent drug ingestion, respectively [4, 5].

TFV-DP in DBS is a validated biomarker of cumulative oral PrEP dosing, given its long intracellular half-life (~ 17 days in red blood cells), reflecting average adherence over the prior 4–8 weeks [4, 5]. FTC-TP, with a short half-life (~ 1.5 days), indicates recent dosing within the prior 2–7 days [4, 5]. Together, TFV-DP and FTC-TP provide a comprehensive assessment of sustained and recent PrEP use, respectively [4, 5].

The most widely validated threshold for TFV-DP is ≥ 700 fmol/punch, corresponding to taking ≥ 4 doses/week, was associated with a near-complete (~ 100%) reduction in HIV acquisition risk in clinical trials [6]. Recent methodological refinements using an updated 50:50 methanol-to-water extraction procedure have recalibrated these thresholds to 900–1599 fmol/punch for 4–6 doses per week [7]. Thus, TFV-DP concentrations of ≥ 700 fmol/punch using the 70:30 extraction method or ≥ 900 fmol/punch using the 50:50 extraction method indicate dosing consistent with ≥ 4 PrEP doses/week and prevention-effective drug exposure among heterosexual men and women [4, 7].

Kenya rapidly scaled oral PrEP delivery through public HIV and reproductive health clinics following national approval in 2015 [8, 9]. Sustaining prevention-effective adherence remains a key priority, as pharmacologic monitoring identified suboptimal TFV-DP concentrations among key populations, such as men who have sex with men (MSM) and adolescent girls and young women (AGYW) [8–11]. These real-world adherence challenges, alongside the contrast between low adherence in early clinical trials and improved adherence in demonstration projects, highlight the importance of objective adherence measurement in routine program settings [12].

Objective measurement of adherence is crucial for interpreting the success of PrEP programs. However, few large-scale evaluations have reported pharmacologic adherence within national PrEP delivery settings. This brief communication provides evidence on adherence to daily oral PrEP using updated TFV-DP and FTC-TP benchmarks from a large implementation project nested within Kenya’s national PrEP program.

## Methods

Data were drawn from the Partners Scale-Up Project (NCT03052010), a stepped-wedge cluster-randomized pragmatic trial conducted between 2017 and 2020 [13]. The trial integrated oral PrEP delivery in 25 real-world public HIV care clinics in Central and Western Kenya, as part of the Kenya National PrEP roll-out program. All clients were HIV-negative adults initiating daily oral PrEP (TDF/FTC; 300 mg/200 mg) [8]. PrEP services, including demand creation, HIV testing, risk assessment, prescribing, counselling, and retention activities were conducted by County Ministry of Health staff as part of standard of care.

Whole-blood samples were obtained from PrEP clients returning for routine refill visits at randomly selected clinics. Each month, a subset of clinics was selected for sampling, and a random subset of scheduled PrEP refill visits (up to 10%) was chosen in advance. Clients attending selected visits were approached for DBS collection. Sampling was therefore restricted to individuals retained in care and returning for refills.

Blood was collected in EDTA tubes and transported to centralized project laboratories. For DBS preparation, 50 µL of whole blood was spotted onto Whatman 903 cards, air-dried for ≥ 3 h (or overnight) at room temperature, stored in plastic bags at − 80 °C, and shipped on dry ice for centralized testing at the University of Colorado. Intracellular TFV-DP and FTC-TP concentrations were extracted from a 3-mm punch using a 50% methanol and 50% water extraction process (50:50) and quantified using validated liquid chromatography-tandem mass spectrometry (LC–MS/MS) methods at the Colorado Antiviral Pharmacology Laboratory (CLIA 06D1094710) [7].

TFV-DP concentrations were classified into weekly dosing categories interpreted according to the recently updated DBS TFV-DP 50:50 benchmarks: ≥1,600fmol/punch for 7 doses/week, 900–1,599 for 4–6 doses/week, 450–899 for 2–3 doses/week, and < 450 for < 2 doses/week [7]. Optimal adherence was defined as ≥ 900 fmol/punch corresponding to ≥ 4 doses/week [7]. FTC-TP concentrations were summarized as the proportion of samples with quantifiable concentrations. Discordant TFV-DP and FTC-TP patterns, though uncommon, were interpreted in line with biomarker half-lives: TFV-DP detection with absent FTC-TP suggested cumulative adherence without very recent dosing, whereas FTC-TP detection with low TFV-DP reflected recent dosing without sustained adherence.

Generalized estimating equations (GEE) regression with Poisson link was used to evaluate the univariate and multivariable association between optimal adherence (≥ 900 fmol/punch) and demographic factors, estimated as risk ratios (RR) and 95% confidence intervals (CI). GEE models used an exchangeable working correlation structure and accounted for within-participant clustering among individuals who contributed multiple DBS samples by clustering on participant ID. Given the limited number of DBS samples, we used a parsimonious modeling approach in which covariates were included in the multivariable model if associated with optimal adherence in univariate analyses (*p* < 0.20). Key demographic variables with strong conceptual relevance to PrEP adherence, including age and sex, were retained a priori in the adjusted model. Statistical significance was considered at *p* < 0.05. Data were analyzed using R software 4.3.2.

## Results

Overall, 4,955 clients initiated PrEP in the large programmatic project. A total of 168 (3%) clients returning for PrEP refills provided 195 randomly collected DBS samples (Supplementary Table S1): 157 participants only had one sample collected, and 11 provided multiple DBS samples. The median (interquartile range [IQR], range) number of samples per client was 1 (1–1, 1–7). The median (IQR) age of the 168 participants who provided samples was of 33 years (27–41); 88% (148/168) were in sero-different relationships, 64% (108/168) were women, 29% (48/168) reported inconsistent or no condom use, and 11% had sexual partner(s) at high risk for HIV and of unknown HIV status.

**Fig. 1:**
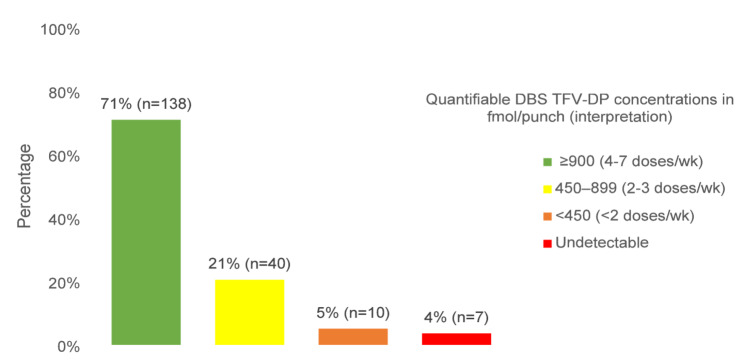
Quantifiable TFV-DP concentrations in DBS samples (*N* = 195). Proportions reflect DBS samples rather than unique participants

The median (IQR) duration between PrEP initiation and sample collection was 5.2 months (3.1–8.7); 13% were on PrEP for 0–2 months at sample collection, 9% for 2–3 months, and 78% for > 3 months. Overall, 96% (188/195) and 88% (172/195) of DBS samples had detectable TFV-DP and FTC-TP, respectively. The median (IQR) concentration for TFV-DP and FTC-TP was 1,247 fmol/punch (829-1,681) and 1,229 fmol/punch (768-1,677), respectively. TFV-DP concentrations consistent with optimal adherence (≥ 900 fmol/punch for TFV-DP) were observed in 71% (138/195) of samples: 32% (63/195) had ≥ 1,600 fmol/punch (corresponding to 7 doses/week), and 39% (75/195) had 900–1,599 fmol/punch (4–6 doses/week) (Fig. 1).

In multivariable analysis, older age (> 25 years) (adjusted risk ratio [aRR] 1.54, 95% CI: 1.05–2.26, *p* = 0.026) and being female (aRR 0.88, 95% CI: 0.82–0.95, *p* = 0.001) were significantly associated with optimal PrEP adherence (≥ 900 fmol/punch) (Table 1).

**Table 1:** Factors associated with optimal PrEP use in the Partners Scale-up project

			Univariate analysis		Multivariable analysis	
	Total *N*	*n* (Row%)	RR (95% CI)	*p* value	aRR (95% CI)	*p* value
**Age**						
<24	35	16 (45.7%)	Reference		Reference	
≥25	160	122 (76.2%)	1.67 (1.09–2.56)	**0.020**	1.54 (1.05–2.26)	**0.026**
**Sex**						
Male	67	53 (79.1%)	Reference		Reference	
Female	128	85 (66.4%)	0.84 (0.78–0.9)	**< 0.001**	0.88 (0.82–0.95)	**0.001**
**Marital status**						
Cohabiting/married	184	132 (71.7%)	Reference			
Single/separated/divorced	11	6 (54.5%)	0.76 (0.48–1.19)	0.234		
**Sex partner(s) at high risk for HIV & HIV status unknown**						
No	172	123 (71.5%)	Reference			
Yes	20	13 (65%)	0.91 (0.76–1.09)	0.291		
**Sero-different relationship**						
No	19	11 (57.9%)	Reference		Reference	
Yes	173	125 (72.3%)	1.25 (0.94–1.65)	0.122	1.20 (0.93–1.55)	0.162
**Inconsistent or no condom use**						
No	123	89 (72.4%)	Reference		Reference	
Yes	69	47 (68.1%)	0.94 (0.86–1.03)	0.171	0.96 (0.88–1.05)	0.344
**Has sex with > 1 partner**						
No	182	131 (72.0%)	Reference			
Yes	10	5 (50.0%)	0.69 (0.37–1.29)	0.250		

(a) Analyses are based on 195 DBS samples contributed by 168 participants

(b) Risk ratios (RRs) were estimated using generalized estimating equations (GEE) regression models with Poisson link

(c) GEE models accounted for within-participant clustering among individuals with repeated samples

(d) aRR = adjusted risk ratio; RR = risk ratio

(e) Covariates included in the multivariable model were selected based on *p* < 0.20 in univariate analyses

(f) Optimal PrEP adherence was defined as TFV-DP ≥ 900 fmol/punch in dried blood spots (50:50 methanol: water extraction), corresponding to ≥ 4 doses/week

## Discussion

In this evaluation of adherence in a real-world PrEP implementation program in Kenya, we observed consistently high objective adherence levels among clients returning for PrEP refills. Nine in ten (96%) of randomly collected DBS samples had quantifiable TFV-DP concentrations and over three-quarters (71%) had concentrations consistent with optimal adherence (≥ 900 fmol/punch). Similarly, FTC-TP detection in most samples (88%) confirmed recent dosing, consistent with the high TFV-DP concentrations that indicate sustained adherence over the preceding weeks. These data demonstrate that optimal adherence (≥ 900 fmol/punch) can be attained among clients with ongoing HIV risk for HIV and engaged in care in real-world PrEP settings.

Our findings align with prior studies from East Africa showing high adherence among PrEP users, particularly in sero-different relationships [14, 15]. While PrEP users in sero-different relationships demonstrate exceptional adherence, other Kenyan populations face significant challenges with PrEP use. Objective adherence measurements reveal stark differences across populations: only 14.5% of MSM achieved optimal adherence levels (≥ 700 fmol/punch), and just 4.6% of AGYW reached this threshold [10, 11]. Women participating in the PrEP Implementation program for Young Women and Adolescents (PrIYA) showed similarly low adherence, with only 30% achieving ≥ 500 fmol/punch [9]. Although most participants in our cohort (88%) were in sero-different relationships, comparisons by relationship status should be interpreted cautiously, given the limited representation of clients outside these partnerships. Nonetheless, the high TFV-DP concentrations observed among clients returning for refills indicate that prevention-effective adherence can be achieved in real-world PrEP delivery settings when individuals perceive ongoing HIV risk.

High TFV-DP concentrations reflect adherence among active PrEP users returning for refills, rather than program-level persistence across all initiators. Early discontinuation remained common in the broader cohort. We previously reported a latent class analysis from the broader program cohort showing that although 44% of clients discontinued PrEP soon after initiation, about one-fourth of clients exhibited consistent high coverage throughout the first year of PrEP use [16]. Hence, highly motivated PrEP clients who prefer oral PrEP and persist beyond the early months can maintain adequate adherence. However, long-acting PrEP options are needed for those who struggle with daily adherence or discontinue it early.

We observed differential demographic patterns in adherence. Older adults and men were more likely to have optimal TFV-DP concentrations (≥ 900 fmol/punch) compared to younger and female PrEP clients. In the PrIYA study, TFV-DP was detected in 72% of women ≥ 24 years compared to 49% of women < 24, indicating lower adherence among younger women [9]. Lower adherence among women may reflect gender-specific structural and relational barriers even within sero-different partnerships, including stigma, constrained autonomy in prevention decision-making, and limited partner support [12, 17]. These barriers may reduce women’s ability to sustain daily pill-taking and consistent engagement in clinic-based prevention services, highlighting the importance of differentiated delivery approaches tailored to women’s needs [12, 17]. These disparities emphasize the need to accelerate access to discreet longer-acting PrEP options alongside targeted adherence support to address persistent gender-related barriers in HIV prevention care [12].

This study had limitations. DBS samples were collected only from clients who returned for PrEP refills, introducing potential selection bias; therefore, the high adherence observed should not be extrapolated to the entire PrEP-initiating population. The pragmatic design may limit generalizability to all public-sector settings. Nevertheless, these findings provide robust real-world evidence of sustained high adherence in routine PrEP delivery.

In conclusion, these findings demonstrate that high adherence to oral PrEP is attainable among clients engaged in care and returning for refills within public-sector HIV prevention programs in Kenya. Strengthening differentiated service delivery approaches and targeted adherence support, particularly for younger clients and women, may help sustain prevention-effective use. As PrEP programs expand, long-acting formulations will be important for individuals who struggle with daily oral adherence or discontinue PrEP early.

## Supplementary Information

Below is the link to the electronic supplementary material.

Supplementary Material 1.

## Data Availability

The datasets used and/or analyzed during the current study are available from the corresponding author on reasonable request.

## Abbreviations

AGYW: Adolescent girls and young women
CI: Confidence intervals
DBS: Dried blood spots
FTC: Emtricitabine
FTC-TP: Emtricitabine triphosphate
GEE: Generalized estimating equations
IQR: Interquartile range
PrEP: Pre-exposure prophylaxis
PrIYA: PrEP Implementation for Young Women and Adolescents
RR: Risk ratio
TDF: Tenofovir disoproxil fumarate
TFV-DP: Tenofovir diphosphate
WHO: World Health Organization
