# Supplementary material for: High adherence to oral daily PrEP in a real-world implementation project in Kenya: a brief communication
Source: AIDS Res Ther. 2026 Mar 24;23:56. doi: 10.1186/s12981-026-00875-4 (PMC13137526; doi:10.1186/s12981-026-00875-4)
Supplement: Supplementary file 1 — Supplementary Material 1. [file 12981_2026_875_MOESM1_ESM.docx]

**Supplementary Table S1. Characteristics of clients who were offered PrEP by the program and who had blood samples collected and tested**

|  | **Total** | **No** | **Yes** |
| --- | --- | --- | --- |
|  |  |  |  |
| N | 4,955 | 4,787 (96.61%) | 168 (3.39%) |
| **Age in years at enrollment** | | | |
| Mean (SD) | 33.29 (10.37) | 33.21 (10.33) | 35.54 (11.27) |
| Median (IQR) | 31 (26-39) | 31 (25-39) | 33 (27-41.25) |
| Missing | 0 (0%) | 0 (0%) | 0 (0%) |
| **Age in years** | | | |
| <24 | 979 (19.76%) | 951 (19.87%) | 28 (16.67%) |
| ≥25 | 3,976 (80.24%) | 3,836 (80.13%) | 140 (83.33%) |
| **Sex** | | | |
| Male | 2,278 (45.97%) | 2,218 (46.33%) | 60 (35.71%) |
| Female | 2,676 (54.01%) | 2,568 (53.65%) | 108 (64.29%) |
| Other | 1 (0.02%) | 1 (0.02%) | 0 (0.00%) |
| **Marital status** | | | |
| Single | 351 (7.08%) | 346 (7.23%) | 5 (2.98%) |
| Cohabiting | 225 (4.54%) | 215 (4.49%) | 10 (5.95%) |
| Married monogamous | 3,718 (75.04%) | 3,588 (74.95%) | 130 (77.38%) |
| Married polygamous | 572 (11.54%) | 554 (11.57%) | 18 (10.71%) |
| Separated/divorced | 72 (1.45%) | 67 (1.40%) | 5 (2.98%) |
| Widowed | 17 (0.34%) | 17 (0.36%) | 0 (0.00%) |
| **Sero-different relationship** | | | |
| No | 817 (16.49%) | 800 (16.71%) | 17 (10.12%) |
| Yes | 4,135 (83.45%) | 3,987 (83.29%) | 148 (88.10%) |
| Missing | 3 (0.06%) | 0 (0.00%) | 3 (1.79%) |
| **Sex partner(s) at high risk for HIV and HIV status unknown** | | | |
| No | 4,152 (83.79%) | 4,005 (83.66%) | 147 (87.50%) |
| Yes | 800 (16.15%) | 782 (16.34%) | 18 (10.71%) |
| Missing | 3 (0.06%) | 0 (0.00%) | 3 (1.79%) |
| **Has sex with >1 partner** | | | |
| No | 4,386 (88.52%) | 4,230 (88.36%) | 156 (92.86%) |
| Yes | 566 (11.42%) | 557 (11.64%) | 9 (5.36%) |
| Missing | 3 (0.06%) | 0 (0.00%) | 3 (1.79%) |
| **Ongoing IPV/GBV** | | | |
| No | 4,916 (99.21%) | 4,752 (99.27%) | 164 (97.62%) |
| Yes | 36 (0.73%) | 35 (0.73%) | 1 (0.60%) |
| Missing | 3 (0.06%) | 0 (0.00%) | 3 (1.79%) |
| **Transactional sex** | | | |
| No | 4,884 (98.57%) | 4,720 (98.60%) | 164 (97.62%) |
| Yes | 68 (1.37%) | 67 (1.40%) | 1 (0.60%) |
| Missing | 3 (0.06%) | 0 (0.00%) | 3 (1.79%) |
| **Recent STI (past 6 months)** | | | |
| No | 4,907 (99.03%) | 4,743 (99.08%) | 164 (97.62%) |
| Yes | 45 (0.91%) | 44 (0.92%) | 1 (0.60%) |
| Missing | 3 (0.06%) | 0 (0.00%) | 3 (1.79%) |
| **Recurrent use of post-exposure prophylaxis (PEP)** | | | |
| No | 4,896 (98.81%) | 4,734 (98.89%) | 162 (96.43%) |
| Yes | 56 (1.13%) | 53 (1.11%) | 3 (1.79%) |
| Missing | 3 (0.06%) | 0 (0.00%) | 3 (1.79%) |
| **Recurrent sex under influence of alcohol/recreational drugs** | | | |
| No | 4,840 (97.68%) | 4,677 (97.70%) | 163 (97.02%) |
| Yes | 112 (2.26%) | 110 (2.30%) | 2 (1.19%) |
| Missing | 3 (0.06%) | 0 (0.00%) | 3 (1.79%) |
| **Inconsistent or no condom use** | | | |
| No | 2,870 (57.92%) | 2,753 (57.51%) | 117 (69.64%) |
| Yes | 2,082 (42.02%) | 2,034 (42.49%) | 48 (28.57%) |
| Missing | 3 (0.06%) | 0 (0.00%) | 3 (1.79%) |
| **Injection drug use (IDU) with shared needles and/or syringes** | | | |
| No | 4,947 (99.84%) | 4,782 (99.90%) | 165 (98.21%) |
| Yes | 5 (0.10%) | 5 (0.10%) | 0 (0.00%) |
| Missing | 3 (0.06%) | 0 (0.00%) | 3 (1.79%) |
|  | | | |
